# A retrospective planning analysis comparing intensity modulated radiation therapy (IMRT) to volumetric modulated arc therapy (VMAT) using two optimization algorithms for the treatment of early-stage prostate cancer

**DOI:** 10.1002/jmrs.22

**Published:** 2013-09-02

**Authors:** Craig A Elith, Shane E Dempsey, Helen M Warren-Forward

**Affiliations:** 1 British Columbia Cancer Agency, Fraser Valley CentreSurrey, BC, Canada; 2 School of Health Sciences, University of NewcastleNewcastle, NSW, Australia

**Keywords:** IMRT, prostate, RapidArc, VMAT

## Abstract

**Introduction:**

The primary aim of this study is to compare intensity modulated radiation therapy (IMRT) to volumetric modulated arc therapy (VMAT) for the radical treatment of prostate cancer using version 10.0 (v10.0) of Varian Medical Systems, *RapidArc* radiation oncology system. Particular focus was placed on plan quality and the implications on departmental resources. The secondary objective was to compare the results in v10.0 to the preceding version 8.6 (v8.6).

**Methods:**

Twenty prostate cancer cases were retrospectively planned using v10.0 of Varian's *Eclipse* and *RapidArc* software. Three planning techniques were performed: a 5-field IMRT, VMAT using one arc (VMAT-1A), and VMAT with two arcs (VMAT-2A). Plan quality was assessed by examining homogeneity, conformity, the number of monitor units (MUs) utilized, and dose to the organs at risk (OAR). Resource implications were assessed by examining planning and treatment times. The results obtained using v10.0 were also compared to those previously reported by our group for v8.6.

**Results:**

In v10.0, each technique was able to produce a dose distribution that achieved the departmental planning guidelines. The IMRT plans were produced faster than VMAT plans and displayed improved homogeneity. The VMAT plans provided better conformity to the target volume, improved dose to the OAR, and required fewer MUs. Treatments using VMAT-1A were significantly faster than both IMRT and VMAT-2A.

Comparison between versions 8.6 and 10.0 revealed that in the newer version, VMAT planning was significantly faster and the quality of the VMAT dose distributions produced were of a better quality.

**Conclusion:**

VMAT (v10.0) using one or two arcs provides an acceptable alternative to IMRT for the treatment of prostate cancer. VMAT-1A has the greatest impact on reducing treatment time.

## Introduction

It is well established that high-dose radical radiation therapy for localized prostate cancer improves disease control.^1^–^4^ Introduced in the early 1990s, three-dimensional conformal radiation therapy (3DCRT) allowed higher doses to be delivered to the prostate and/or planning target volume (PTV), and acceptable dose to be delivered to surrounding healthy tissues compared to previous methods.^5^ However, since the mid-2000s, intensity modulated radiation therapy (IMRT) has become the standard technique to deliver external beam radiation therapy treatment to the prostate, due to its increased ability to deliver higher dose treatment to the PTV while reducing dose to the surrounding critical organs and healthy tissues.^6^,^7^ Standard IMRT approaches achieve this through the use of multiple fixed gantry radiation fields which each deliver irregular intensity patterns of dose across the PTV in response to preset plan objectives that when summed together provide highly conformal dose distributions within and across a PTV.

The improved dose distribution achieved using standard IMRT comes with a cost of longer treatment times due to increased set-up and verification methods and increased monitor units (MUs).^8^ The longer treatment time using IMRT can lead to increased patient discomfort, reduced machine throughput, and an increased chance of geographical target miss due to patient movement.^7^ Increasing the number of MUs results in a greater integral body dose from leakage and scatter radiation, increasing the risk of developing a secondary malignancy.^9^

In 2008, Otto reported a novel form of IMRT called volumetric modulated arc therapy (VMAT).^10^ In VMAT, treatment is delivered using a cone beam that rotates around the patient. The cone beam is modulated by the intertwining of dynamic multileaf collimators (MLCs), variable dose rates, and gantry speeds to generate IMRT quality dose distributions in a single optimized arc around the patient.^11^

There is a growing body of literature supporting that VMAT is capable of delivering treatment to the prostate with a similar or better dose distribution compared to fixed-field IMRT, yet requires significantly fewer MUs and reduced treatment time than IMRT.^6^–^8^,^12^–^23^

In 2010, the Fraser Valley Centre (FVC) of the British Columbia Cancer Agency (BCCA) considered implementing VMAT utilizing Varian Medical System's (Palo Alto, CA) *RapidArc*. To assess the degree to which the VMAT technology at FVC could provide for efficient and effective planning outcomes, the authors of this study undertook research which compared a 5-field sliding window IMRT technique (the standard technique at FVC for prostate treatment) to VMAT using either one or two treatment arcs.^24^ This research was done using version 8.6 (v8.6) of the *RapidArc* (VMAT) planning software, which was at this time the clinical planning system in use at FVC. Particular emphasis was placed on the utilization of planning and treatment resources. From this research it was concluded that VMAT demonstrated the ability to have increased treatment efficiency, as well as requiring fewer MUs to deliver a single treatment fraction. However, v8.6 was unable to achieve departmental planning guidelines for all the plans tested when using a single arc. Also, extended time was needed to generate the VMAT plans compared to standard IMRT plans. The FVC therefore continued to use IMRT for the radical treatment of early prostate cancer in v8.6 of the planning software.

In October 2011, FVC upgraded to version 10.0 (v10.0) of the Varian's *RapidArc* (VMAT) system. The most significant difference between v8.6 and v10.0 of the *RapidArc* (VMAT) planning software is in the progressive resolution optimizer algorithm (PRO). v8.6 uses PRO8.6.15, whereas v10.0 uses PRO10.0.28. It is beyond the scope of this article to detail the differences between the PRO algorithm utilized in v8.6 and v10.0, which has been reported elsewhere.^25^ For the purposes of this article, it suffices to say that in v10.0, the PRO algorithm has been modified and it is suggested that the newer version is able to generate plans of improved quality in less time than the version of PRO utilized in v8.6.^25^

In the research presented within this study, IMRT and VMAT will be compared for the treatment of early-stage prostate cancer using v10.0 of the *RapidArc* (VMAT) software. Emphasis will be placed on the utilization of planning and treatment resources, while also examining the quality of the treatment plans being produced. Comparisons will also be made between the outcomes obtained previously in v8.6 and the upgraded v10.0 to assess if sufficient improvements have been made in the VMAT process to reconsider utilizing this technique to routinely treat prostate cancer at our department.

## Materials and Methods

Approval for this study was provided by the University of Newcastle, Australia, Human Research Ethics Committee (approval number: H-2011-0073), and the British Columbia Cancer Agency, Canada, Research Ethics Board (approval number: H11-00108).

Full details of the materials and methods used in this study have been reported previously in a study describing our experiences using v8.6 of Varian Medical System's *RapidArc* (VMAT) software.^24^ The previously described methods have been reproduced here to detail our experience using v10.0 of the software.

### Cases and plans

**Figure 1:**
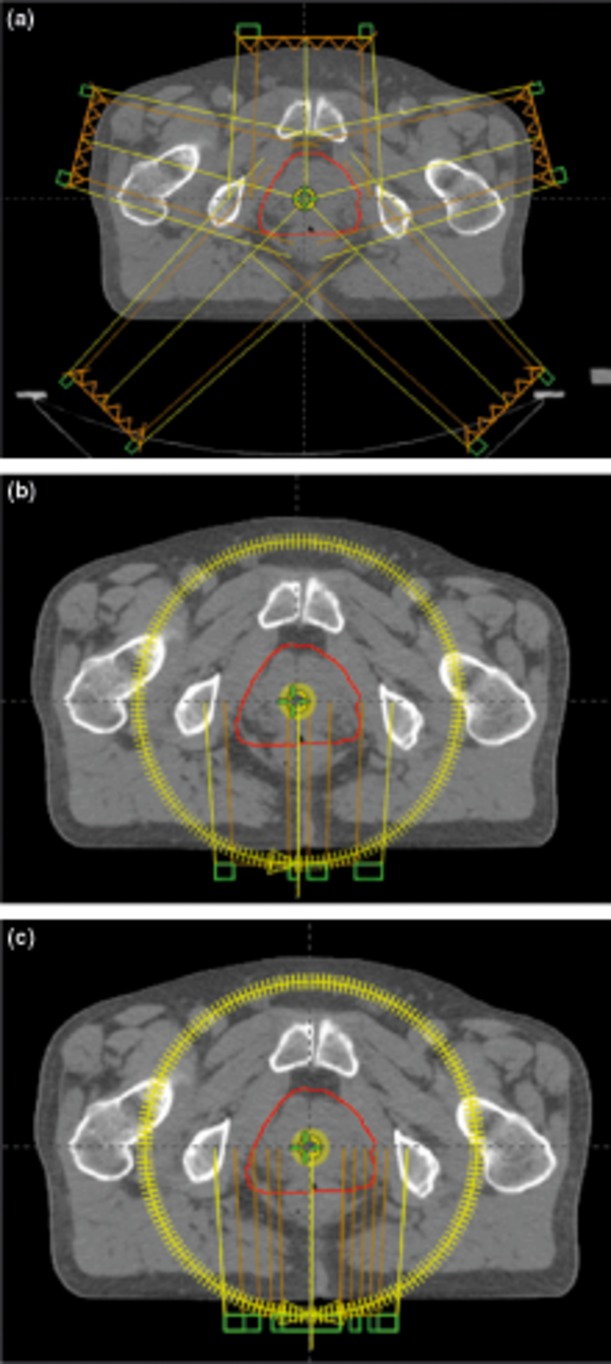
An example case displaying the planning target volume (in red) and the beam arrangement for (A) 5- field intensity modulated radiation therapy (IMRT), (B) volumetric modulated arc therapy (VMAT) using one arc (VMAT-1A) and (C) VMAT using two arcs (VMAT-2A).

The study used deidentified CT data sets from 20 patients who had been previously treated at FVC with IMRT to the prostate only. Dose distributions were generated retrospectively for each data set using three techniques: a 5-field sliding window IMRT, VMAT using one full gantry rotation (VMAT-1A), and VMAT with two complete arcs in opposite directions (VMAT-2A) (Fig. 1). All planning was done by the same radiation therapist using v10.0 of Varian Medical System's *Eclipse* planning software (which includes *RapidArc)*. All planning was done on the same computer which uses an XP (SP3) operating system, 16 processors (2.3 GHz each), and 24 GB of RAM. Each plan was prescribed 7400 cGy in 37 fractions and intended to meet the FVC prostate IMRT planning guidelines outlined in Table 1.

**Table 1:** The Fraser Valley Centre–specific planning objectives for both the intensity modulated radiation therapy (IMRT) and volumetric modulated arc therapy (VMAT) treatments of the prostate

Volume/organ at risk (OAR)	Dose constraint
Planning target volume (PTV)	99% of the volume to get ≥95% of the prescription
	Minimum dose >90% of the prescription
	Mean dose >99% of the prescription
	Maximum dose <107% of the prescription
	The maximum dose must be within the PTV
Rectum	<65% of the volume to receive 50 Gy
	<55% of the volume to receive 60 Gy
	<25% of the volume to receive 70 Gy
	<15% of the volume to receive 75 Gy
	<5% of the volume to receive 78 Gy
Bladder	<50% of the volume to receive 65 Gy
	<35% of the volume to receive 70 Gy
	<25% of the volume to receive 75 Gy
	<15% of the volume to receive 80 Gy

Gy, dose in gray.

### CT simulation

The original CT data sets were obtained on a Phillips Brilliance Big Bore scanner using 2-mm slices with the patient in a supine position. Patients were instructed to have a full bladder at time of simulation and treatment; however, bowel preparation to ensure an empty bowel was not performed.

### Contouring

All original contours from the actual treatment plans were transferred onto the deidentified data sets.

A radiation oncologist contoured the prostate, bladder, and rectum from the sigmoid colon to the anus. A PTV was generated by expanding the prostate contour with a 10-mm margin in all directions. If the data set included prostate fiducial markers, the PTV was created using a 6-mm margin to the prostate posteriorly to spare additional rectal tissue from receiving radiation dose.

Optimization structures were created for the PTV, rectum, and bladder. A PTV_opti_ was created by copying the PTV and extending the contour superiorly and inferiorly by one slice. The size of the PTV_opti_ on the new superior and inferior slices was reduced by half. The creation of the PTV_opti_ was done to allow the superior and inferior ends of the PTV to receive adequate dose coverage via primary and scatter dose. Rectum_opti_ and Bladder_opti_ structures were created by subtracting the rectum and bladder structures from the PTV_opti_ plus a 3-mm margin.

In addition to the contours transferred from the original planning data, the heads of femur were also contoured. The dose to the heads of femur is not routinely considered for IMRT planning at FVC, but was considered in this study. The heads of femur were contoured superiorly from the caudal ischial tuberosity.

A couch structure was added to the plans so that beam attenuation from the treatment couch was considered. The couch structure was added using the predefined couch structures available within the Varian's *Eclipse* software.

### IMRT

At our centre, a 5-field sliding window IMRT technique is standardly used to treat the prostate. A template is used to expedite the planning process. The template defines the gantry angles of the 5 treatment fields as well as the optimization parameters. Each treatment beam uses 6-MV photons with the gantry angles fixed at 0°, 75°, 135°, 225°, and 285° (Fig. 1A). Dosimetric calculations were performed using the anisotropic analytical algorithm (AAA) with heterogeneity correction on and a 2.5-mm calculation grid.

### VMAT

In this study, both a single-arc and two-arc VMAT plan were developed. Similar to IMRT, plan templates defining beam parameters and the initial optimization objectives were created to expedite the planning process. Importantly, the initial optimization objectives used for VMAT planning were different to those set for IMRT. The same optimization template was utilized for both VMAT techniques; however, these objectives were adjusted during optimization to achieve the best plan.

The single-arc technique (VMAT-1A) utilized one complete counterclockwise (CCW) rotation to deliver radiation treatment (Fig. 1B). The gantry start angle was 179° and the stop angle was 181°. The collimator was set at 45° to minimize MLC tongue and groove effect.^13^

The two-arc plan (VMAT-2A) combined both a complete CCW rotation and a full clockwise (CW) gantry rotation for treatment (Fig. 1C). The parameters for the first arc were identical to the VMAT-1A technique. The second arc had the gantry rotating in the opposite direction to minimize set-up time. The gantry start angle was 181° and the stop angle was 179°. For the two-arc plan, the collimator rotation was set to 135° to increase modulation. VMAT calculations utilized AAA with heterogeneity correction on and a 2.5-mm calculation grid.

### Analysis

#### Plan quality

A dose distribution was considered acceptable for treatment if able to meet the FVC prostate IMRT planning guidelines (Table 1).

The plan quality was quantitatively assessed by calculating the homogeneity index (HI) and conformity number (CN) for each plan. The HI is defined as


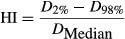


where *D*_*n*_ is the dose covering *n* of the target volume**.**

A HI value closer to zero indicates more homogeneous dose coverage within the PTV.

Dose conformity evaluates the dose fit of the PTV relative to the volume covered by the prescription dose.^17^ Ideally the prescribed dose should fit tightly to the target volume, therefore reducing the side effects occurred by treating surrounding tissues and organs. The CN simultaneously takes into account irradiation of the target volume and irradiation of healthy tissues.^26^ The CN is defined as


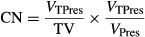


where *V*_Pres_ is the total volume receiving the prescription, *TV* is the target volume, and *V*_TPres_ is the target volume covered by the prescription.^27^

A CN value closer to 1 indicates that the dose distribution fits more tightly to the target volume preserving healthy tissue.

#### Dose to organs at risk

The dose to organs at risk (OAR) was compared by determining the percentage volume (*V*) of an organ receiving *n* dose (*V*_*n*_). To get a complete understanding of how IMRT and VMAT planning impacts on dose delivered across the rectum and bladder, the *V*_5_, *V*_15_, *V*_20_, *V*_30_, *V*_40_, *V*_50_, *V*_60_, *V*_65_, and *V*_70_ were recorded. For each of the left and right heads on femur, the *V*_30_ and *V*_40_ were measured.

#### Planning time

The time taken to generate a dose distribution for each technique was recorded. For the purposes of this study, planning time does not include the time needed to perform contouring as this is considered neutral for both IMRT and VMAT planning. Instead, time measurement includes a sum of the time to place fields, plan optimization, dose calculation, and the period of evaluation of the final dose distribution to assess if the planning guidelines were achieved.

#### Treatment time

The time taken to treat the IMRT, VMAT-1A, and VMAT-2A plans was measured and recorded. This was done by running the treatment plan for all three techniques in stand-by mode on a Varian Trilogy linear accelerator. Time measurement was started at the initial beam-on and was ended when the final MU was delivered. The treatment time does include the time taken to move parameters such as gantry and collimator angles during treatment and between fields. The measured treatment time does not include patient set-up time or the time that may be needed to verify treatment position.

#### Number of MUs

The total number of MUs needed to deliver each treatment plan was summed and recorded.

#### Comparing v8.6 to v10.0

The results of the planning of the 20 cases using v10.0 of the planning software were compared to the previously reported results using v8.6.^24^

### Statistical analysis

A sample size of 20 cases was calculated to give a power of at least 0.8 at the 95% level. Statistical analysis was conducted using Graphpad InStat version 3 for windows (http://www.graphpad.com). The data were analysed first to test for normality, and if it passed it was analysed for statistical difference with the parametric paired *t*-test and repeated measures analysis of variance (RM ANOVA). If the data were not normal, then statistical difference was analysed using Wilcoxon matched-pairs and the Friedman test (nonparametric repeated measures ANOVA). A paired test was chosen as the same data sets were used for each treatment option. To be statistically different, the values were needed to be significant at the 95% level (i.e., *P* < 0.05).

## Results

Using v10.0, a dose distribution that met the planning guidelines was able to be produced for each of the IMRT, VMAT-1A, and VMAT-2A techniques at the first attempt. The overall quality of the plans produced was similar; however, statistically significant differences were noted among the three techniques.

The results for HI, CN, planning time, treatment time, and number of MUs using v10.0 of the planning software are presented in Table 2.

**Table 2:** Summary data representing the median planning time, treatment time, MUs required, homogeneity index, and conformity number for the IMRT, VMAT-1A, and VMAT-2A plans using version 10.0 of the Varian Medical System's *RapidArc*

	Median (95% confidence interval)			*P*-values			
		
	IMRT	VMAT-1A	VMAT-2A	RM ANOVA	IMRT versus VMAT-1A	IMRT versus VMAT-2A	VMAT-1A versus VMAT-2A
Planning time (min)	9.75 (9.14–10.12)	18.4 (17.95–19.47)	18.42 (17.52–19.49)	<0.001	<0.001	<0.001	0.35*
Treatment time (min)	3.14 (3.11–3.27)	1.3 (1.29–1.31)	3.18 (3.16–3.19)	<0.001	<0.001	0.64*	<0.001
Monitor units	594.0 (578.3–638.8)	446.5 (436.5–461.9)	450.5 (442.0–464.4)	<0.001	<0.001	<0.001	0.27*
Homogeneity index	0.0385 (0.036–0.042)	0.065 (0.062–0.066)	0.061 (0.059–0.063)	<0.001	<0.001	<0.001	0.018
Conformity number	0.748 (0.73–0.76)	0.843 (0.84–0.845)	0.851 (0.84–0.85)	<0.001	<0.001	<0.001	0.009

IMRT, 5-field sliding window intensity modulated radiation therapy; VMAT-1A, volumetric modulated arc therapy using one full arc; VMAT-2A, volumetric modulated arc therapy using two full arcs.

* Illustrates where a significant difference was NOT observed.

Conformity of the dose to the PTV (CN) is significantly better for both VMAT plans than IMRT. The median CN for VMAT-2A is better than that for VMAT-1A, although there is no statistically significant difference between the two VMAT techniques.

The dose uniformity (HI) across the PTV is significantly better for the IMRT dose distributions compared to both VMAT techniques. The median HI for VMAT-2A is better than that for VMAT-1A, although not statistically significant.

IMRT plans were produced in a median time of 9.7 min. This was significantly faster than the VMAT-1A and VMAT-2A techniques, which required twice as long to generate (18.4 and 18.4 min, respectively).

VMAT-1A treatments were performed in 1.3 min. This was less than half the time needed for both VMAT-2A and IMRT treatments which were similar in treatment time (3.2 and 3.1 min, respectively).

Both VMAT techniques required a similar number of MUs to deliver a single fraction of treatment. VMAT-1A required a median of 446.5 MUs, whereas VMAT-2A used 450.5 MUs. IMRT required significantly more MUs (594) to deliver a single treatment.

A comparison of HI, CN, planning time, treatment time, and number of MUs between v8.6 and v10.0 is presented in Table 3. In the comparison between v8.6 and v10.0 there are two outstanding items. First, when planning VMAT-1A in v8.6, the planning guidelines were only achieved in 8 of the 20 data sets, whereas in v10.0, the VMAT-1A technique was able to successfully meet the same planning guidelines for each of the same 20 data sets. Second, the time needed to generate VMAT-1A and VMAT-2A plans is significantly reduced in v10.0.

**Table 3:** Comparison of version 8.6 (v8.6) to version 10.0 (v10.0) of the Varian Medical System's *RapidArc*

	IMRT			VMAT-1A			VMAT-2A		
			
	v8.6 median (*N* = 20)	v10.0 median (*N* = 20)	*P*-value	v8.6 median (*N* = 8)	v10.0 median (*N* = 20)	*P*-value	v8.6 median (*N* = 20)	v10.0 median (*N* = 20)	*P*-value
Planning time (min)	9.86	9.75	0.357*	30.57	18.40	<0.0001	43.92	18.42	<0.0001
Treatment time (min)	3.18	3.14	0.001	1.34	1.30	<0.0001	3.28	3.18	<0.0001
Monitor units	600.5	594.0	0.016	511.5	446.5	0.0004	557	450.5	<0.0001
Homogeneity index	0.0365	0.0385	0.247*	0.0655	0.0650	0.376*	0.0455	0.0610	0.0001
Conformity number	0.791	0.748	<0.0001	0.827	0.843	0.028	0.815	0.851	<0.0001

Compared endpoints include median planning time, treatment time, monitor units required, homogeneity index, and conformity number for IMRT, VMAT-1A, and VMAT-2A plans. IMRT, 5-field sliding window intensity modulated radiation therapy; VMAT-1A, volumetric modulated arc therapy using one full arc; VMAT-2A, volumetric modulated arc therapy using two full arcs.

* Illustrates where a significant difference was NOT observed.

The doses delivered to the OARs using v10.0 are presented in Table 4. VMAT is demonstrated to deliver lower dose than IMRT to the bladder and heads of femur. Likewise, the dose delivered to the rectum in the *V*_60_–*V*_70_ range is improved using VMAT. In the *V*_20_–*V*_30_ range, IMRT delivers a lower dose to the rectal tissue.

**Table 4:** The dose to the rectum, bladder, and heads of femur observed using version 10.0 of the Varian Medical System's *RapidArc*. The dose to the organ at risk is presented as the percentage volume (*V*) of the organ receiving *n* dose in gray (*V*_*n*_)

	IMRT		VMAT-1A		VMAT-2A		*P*-values (*N* = 20)		
				
	Median (%)	95% confidence interval	Median (%)	95% confidence interval	Median (%)	95% confidence interval	IMRT versus VMAT-1A	IMRT versus VMAT-2A	VMAT-1A versus VMAT-2A
Rectum									
*V*_5_	94.2	86.1–95.1	93	86.0–95.0	93.4	86.3–95.2	0.32	0.71	0.025*
*V*_15_	77.7	71.5–83.6	78.4	70.8–82.7	78.4	70.7–82.7	0.19	0.2	0.75
*V*_20_	69.1	62.8–75.9	75.1	67.8–79.6	75.3	67.8–79.7	0.002*	0.003*	0.89
*V*_30_	60.3	54.1–66.7	65.5	58.9–68.2	65.1	59.1–67.5	0.03*	0.07	0.65
*V*_40_	48.6	40.2–51.6	48.8	44.9–53.2	48	44.8–52.1	0.02*	0.06	0.27
*V*_50_	31.1	27.2–36.4	31.6	29.2–37.1	30.9	28.7–36.3	0.01*	0.2	<0.001*
*V*_60_	23	19.2–27.6	21	18.6–26.4	21.1	18.5–26.3	0.01*	0.01*	0.51
*V*_65_	18.9	15.5–23.2	17.1	14.5–21.8	17.1	14.6–21.8	<0.01*	<0.001*	0.72
*V*_70_	14.1	11.6–18.3	11.9	10.2–16.5	12.1	10.2–16.5	<0.001*	<0.001*	0.92
*V*_75_	1.3	0.8–2.8	0.8	0.7–3.6	0.4	0.5–2.5	0.77	0.35	<0.001*
Bladder									
*V*_5_	64.3	58.4–78.6	66.1	59.5–79.5	66.15	59.4–79.5	0.03*	0.03*	>0.99
*V*_15_	47.2	41.4–65.3	44.7	39.3–63.3	45.7	39.3–63.2	<0.001*	<0.001*	0.95
*V*_20_	43.4	38.1–61.6	39.3	35.7–59.3	39.4	35.7–59.3	<0.001*	<0.001*	0.8
*V*_30_	33.5	30.7–52.3	29.4	28.7–51.0	30.8	28.6–50.7	0.003*	<0.001*	0.47
*V*_40_	24.2	22.1–39.7	22.5	22.1–41.2	23.4	22.0–40.9	0.39	0.83	0.35
*V*_50_	19.2	18.1–32.9	17.3	16.9–32.6	17.2	16.9–32.3	0.04*	0.006*	0.44
*V*_60_	15.4	14.7–27.1	13	13.1–25.6	13.1	13.1–25.5	<0.001*	<0.001*	>0.99
*V*_65_	13.4	12.9–24.1	11.2	11.4–22.4	11.3	11.4 -22.3	<0.001*	<0.001*	0.78
*V*_70_	10.9	10.8–20.4	9.1	9.3–18.5	9.2	9.3–18.5	<0.001*	<0.001*	0.33
*V*_75_	3.5	2.9–7.8	23	2.1–5.2	2.2	19.–4.6	0.04*	0.013*	0.116
Left femur									
*V*_30_	25.8	21.0–33.1	1.5	2.2–11.3	0.5	0.4–5.8	<0.001*	<0.001*	<0.001*
*V*_40_	7.3	4.9–12.0	0	0–0.9	0	0–0.9	<0.001*	<0.001*	0.19
Right femur									
*V*_30_	29.1	23.9–36.8	2.5	1.3–10.9	1	1.2–7.9	<0.001*	<0.001*	0.33
*V*_40_	11.5	8.2–17.2	0	0–1.3	0	0–0.9	<0.001*	<0.001*	0.4

IMRT, 5-field sliding window intensity modulated radiation therapy; VMAT-1A, volumetric modulated arc therapy using one full arc; VMAT-2A, volumetric modulated arc therapy using two full arcs; *V*_*n*_, The percentage volume (*V*) of an organ receiving *n* dose.

* Illustrates where a significant difference was observed.

## Discussion

In the first part of this study, which sought to evaluate the differences between IMRT and VMAT techniques using v10.0 software, each technique was able to generate a dose distribution that was adequate for treatment. The overall quality of the plans produced were similar; however, statistically significant differences were noted among the three techniques.

The dose uniformity across the PTV reported by the HI is significantly better for IMRT than both VMAT techniques. Others have reported a similar trend for homogeneity.^6^,^15^,^16^,^20^,^24^ The lower homogeneity is reported to be inherent to the optimization algorithm used for VMAT planning.^6^,^10^

Volumetric modulated arc therapy planning was demonstrated to produce dose distributions that had a better conformity to the PTV than IMRT. This outcome supports the findings from previous published research.^13^,^16^,^20^,^24^ The improved conformity observed using VMAT is a consequence of arc delivery that delivers dose from 360°. The improvement in dose conformity observed using VMAT may increase the potential of dose escalation without increasing treatment-related morbidities associated with radiation exposure to surrounding tissues. Dose escalation has been demonstrated to improve local control of prostate cancer.^1^–^4^,^12^ Despite demonstrating improved conformity to the PTV, dose escalation using VMAT may still be limited by planning hotspots that have been reported to be greater for VMAT than IMRT.^6^,^15^,^20^

There is a growing body of evidence supporting that VMAT treatment of prostate cancer is significantly faster and requires fewer MUs compared to IMRT.^6^–^8^,^12^–^23^ As expected, our results demonstrate that the treatment time using the VMAT-1A technique was significantly faster than using IMRT. The reduced treatment time of VMAT-1A means there is less patient discomfort during treatment and a reduced risk of patient movement. The reduced treatment time may also prove to be biologically advantageous. Evidence has shown that the radiation survival is not only a function of the total dose delivered but also depends on the duration that the radiation is delivered.^28^,^29^ There is a potential tumour cell killing benefit to deliver radiation doses in a shorter time.^30^

The reduced treatment time using VMAT-1A also holds enormous resource potential. The faster treatments could allow more patients to receive treatments daily and therefore reduce waitlists. Alternatively, the extra time available on a treatment unit can be utilized to implement advanced image-guided radiation therapy (IGRT) protocols or implement advanced treatment techniques for other treatment sites that require longer treatment times, without increasing waitlists.

It is important to note that there was no significant difference in the treatment times needed for the IMRT and VMAT-2A techniques. This result demonstrates that the treatment time advantage VMAT offers is reduced when using more than one arc for treatment.

Also as expected, it was demonstrated in this study that the VMAT plans required fewer MUs to deliver a fraction of treatment. The decrease in MUs required for VMAT treatments reduces a patient's exposure to scatter and leakage radiation, which is a concern regarding the development of secondary cancers.^9^,^31^ Secondary malignancy induction is a more important consideration as ongoing technical improvements in cancer diagnosis and treatment are improving the prognosis for patients being treated with radiation.^23^

The median dose to the bladder was lowered with VMAT for all measured volumes. Similarly, the dose delivered to the rectum in the *V*_60_–*V*_70_ range is improved using VMAT. These results are in compliance with VMAT demonstrating improved conformity to the PTV than IMRT. In the *V*_20_–*V*_30_ range, IMRT delivers a lower dose to the rectal tissue. The observed doses to the rectum may be explained in that the IMRT technique selects angles that avoid the rectum, whereas the VMAT techniques may distribute lower dose through the rectum to achieve conformal coverage of the PTV in the high-dose region. This phenomenon is not observed in the bladder as it mostly sits superior to the PTV.^6^

It was demonstrated here that the median dose to the heads of femur was lower using VMAT compared to IMRT. It is important to note that in this study constraints were not applied to the heads of femur during optimization for either the VMAT or IMRT plans. If constraints were applied, it would be reasonable to expect that the dose delivered to the heads of femur would be further reduced. Others have reported that when constraints are applied to the heads of femur during optimization, VMAT delivers a lower dose than IMRT to these structures.^6^,^8^,^20^

In the second part of this research, which compared IMRT and VMAT plans developed with v8.6 to v10.0 software, the outcomes demonstrate that the transition to more advanced planning and treatment methods need to be implemented in line with the integration of the appropriate software.

In v10.0, plans suitable for treatment were produced for each of the 20 data sets using VMAT-1A. Importantly, these dose distributions achieved the planning guidelines at the first attempt. In contrast, using v8.6, VMAT-1A was capable of producing plans that achieved the planning guidelines for only eight of the 20 data sets. Notably, the guidelines were achieved at the first attempt for only two of the eight cases.^24^

In addition, VMAT plans were able to be generated using v10.0 in a fraction of the time required in v8.6. In the latest version of the software, IMRT plans were generated in a median time of 9.75 min, whereas both VMAT techniques required approximately 9 min longer to be produced. Statistically, the additional 9 min needed to generate a VMAT plan is significant; however, it may be argued that this time is clinically insignificant within the planning module. The additional minutes needed to produce a VMAT plan instead of an IMRT distribution is only a small fraction of the overall planning time when you also consider contouring times and quality assurance checks.

The improvements observed in v10.0 have significant resource implications in the utilization of VMAT clinically. Previously it was concluded that our department would not implement VMAT (v8.6) for the treatment of prostate cancer due to the inability to achieve the departmental planning guidelines and significantly prolonged planning time. In v10.0, the uncertainty of achieving the planning guidelines with VMAT is eliminated. Also the additional time needed to produce the VMAT plans has been reduced significantly and may now be considered of no consequence clinically.

## Conclusion

It has been demonstrated that using v10.0 of Varian Medical System's *Eclipse* and *RapidArc* (VMAT) software, a dose distribution that meets our departmental planning guidelines can be generated using IMRT, VMAT-1A, and VMAT-2A. The overall quality of the plans produced were similar; however, statistically significant differences were noted among the three techniques. Importantly, treatment times are reduced when using VMAT-1A, and the number of MUs required to deliver a fraction of treatment is lower for VMAT than IMRT.

Based on these findings our department is considering implementing VMAT for the radical treatment of prostate cancer to take advantage of the reduced treatment time and the reduced number of MUs. Future directions will include considering the resource implications of using VMAT-1A versus VMAT-2A or perhaps utilizing partial arcs to get the best mix of plan quality and utilization of departmental resources.
